# Can MLVA Differentiate among Endemic-Like MRSA Isolates with Identical *Spa*-Type in a Low-Prevalence Region?

**DOI:** 10.1371/journal.pone.0148772

**Published:** 2016-02-09

**Authors:** Anita Blomfeldt, Abdullahi Abdi Hasan, Hege Vangstein Aamot

**Affiliations:** 1 Department of Microbiology and Infection Control, Akershus University Hospital, Lørenskog, Norway; 2 Faculty of Health Sciences, Oslo and Akershus University College of Applied Sciences, Oslo, Norway; 3 Department of Clinical Molecular Biology (EpiGen), Division of Medicine, Akershus University Hospital and University of Oslo, Lørenskog, Norway; The University of Hong Kong, HONG KONG

## Abstract

The prevalence of methicillin-resistant *Staphylococcus aureus* (MRSA) in Norway is low, but an endemic-like MRSA clone with Staphylococcal protein A (*spa*)-type t304 has been established especially in nursing homes in the Oslo region causing several large outbreaks. The challenge was that *spa*-typing and the gold standard Pulsed-Field Gel Electrophoresis (PFGE) were inadequate in discriminating isolates in outbreak investigations. Additional higher resolution genotyping methods were needed. The aims of this study were a) to evaluate whether Multiple-Locus Variable number of tandem repeat Analysis (MLVA) could differentiate within the PFGE clusters between epidemiologically related and unrelated endemic-like ST8-MRSA-IV-t304-PVL-neg (MRSA-t304) isolates and b) investigate the evolution of the endemic-like MRSA-t304 clone over a 15-year time period. All MRSA-t304 isolates detected in the region from 1998 through April 2013 were included. In total, 194 of 197 isolates were available for PFGE and MLVA analyses. PFGE results on isolates from 1998–2010 have been published previously. Two PFGE clusters subdivided into eight MLVA types were detected. One major outbreak clone (PFGE cluster C2/ MLVA type MT5045) appeared from 2004 to 2011 causing long-lasting and large outbreaks in seven nursing homes and one hospital. Five new MLVA types (N = 9 isolates) differing in only one VNTR compared to the outbreak clone C2/MT5045 were detected, but only one (C2/MT5044) was seen after 2011. We suggest that MLVA can replace PFGE analysis, but MLVA may not be the optimal method in this setting as it did not discriminate between all epidemiologically unrelated isolates. The results may indicate that all eight outbreaks in different locations within the PFGE C2 cluster may be branches of one large regional outbreak. The major outbreak strain C2/MT5045 may now, however, be under control, extinguished or has moved geographically.

## Introduction

Norway has a “search-and-destroy” policy against methicillin-resistant *Staphylococcus aureus* (MRSA) [1]. The aim is to limit the spread of antibiotic resistance and keep MRSA out of hospitals and other healthcare institutions. Still, our recent publication demonstrated establishment of an endemic-like MRSA clone with Staphylococcal protein A (*spa*-) type t304 (ST8-MRSA-IV-t304-PVL-negative (MRSA-t304)) especially in nursing homes in the South-Eastern parts of Norway [2]. This clone posed a challenge in surveillance and outbreak investigations because not only did the bacteria require extraordinary culturing conditions, but *spa*-typing and gold-standard typing method Pulsed-Field Gel Electrophoresis (PFGE) were inadequate typing methods in discriminating between epidemiologically related and unrelated isolates [2].

Among the 180 MRSA-t304 isolates from a 13-year period, PFGE could only differentiate the isolates into two clusters [2]. In this setting, the need for additional typing methods is apparent as the isolates represented 9 outbreaks in different locations and several sporadic cases with and without connection to healthcare settings and a long evolutionary time span.

Determining if there is an outbreak or not is of great importance as outbreaks initiates several measures including tracing infection source and infected or colonized individuals through systematic screening procedures, possible isolation and decolonization of individuals with MRSA, and other efforts that can limit cross-contamination and further spread.

Next generation sequencing is on the rise in surveillance and outbreak investigations of MRSA [3–7], but more insight is needed in how to interpret genetic relatedness between isolates and improved software pipelines designed for diagnostic use is required [7]. Multiple-Locus Variable number of tandem repeat Analysis (MLVA) with eight VNTR (Variable Number of Tandem Repeat) loci [8] was therefore applied. This protocol has been well validated and meet the consensus criteria of typing methods [9]. In addition to having a uniform nomenclature (www.mlva.net), MLVA’s discriminatory power has been shown to be at least as good as PFGE and superior to *spa*-typing as well as being capable of discriminating within identical *spa*-types [8, 10].

The aims of this study were a) to evaluate whether MLVA could differentiate within the PFGE clusters between epidemiologically related and unrelated endemic-like MRSA-t304 isolates and b) investigate the evolution of the endemic-like MRSA-t304 clone over a 15-year time period. Our results support that MLVA can replace PFGE and demonstrated several different, but closely related MRSA-t304 MLVA types and one major outbreak clone in 2004–2011.

## Material and Methods

Akershus University Hospital (Ahus), Lørenskog, Norway performed genotyping of *S*. *aureus*, including molecular outbreak investigations, for Oslo and Akershus counties from 2000 up to 1. May 2013. This region constituted ~25% of the Norwegian population.

All MRSA-t304 isolates detected in the region and genotyped at Ahus from 1998 through April 2013 were eligible for inclusion. Only the first isolate per patient was included. Place and date of sampling were recorded. The isolates were defined as sporadic cases or belonging to an outbreak based on available epidemiological data. An outbreak was defined as two or more cases with the same *spa*-type epidemiologically linked through individual, time and space. A sporadic case was defined as having no epidemiological link to any known outbreak. Nomenclature regarding isolate origin and PFGE clusters was adapted from our previous publication [2].

MLVA and PFGE were performed on all isolates. The MLVA protocol [8] was modified with desalted primers and run on a 3130xl Genetic Analyzer (Applied Biosystem) with prolonged run-time to 8400 seconds. BioNumerics software version 7.1 (Applied Maths, Belgium) was used in calculation of fragment sizes, MLVA profiles and creation of minimum spanning tree using default settings. The MLVA profiles were assigned to MLVA types (MT) using the profile query at www.mlva.net.

Isolates from 1998–2010 (N = 177) had previously been typed by PFGE [2] whereas the remaining 17 isolates were typed in this study. The Harmony protocol was followed in both cases [11]. The PFGE profiles were analysed and clustered with BioNumerics software version 7.1 using the Dice coefficient and UPGMA with 1.25% band position tolerance and optimization of 0.5%. PFGE patterns with ≥80% similarity were assigned to PFGE clusters.

Simpson’s index of diversity (SID) was used to evaluate the discriminatory power of MLVA and PFGE and was calculated using http://darwin.phyloviz.net/.

The study was approved by the local Data Protection Official (ref. no. 14-002).

## Results

**Fig 1:**
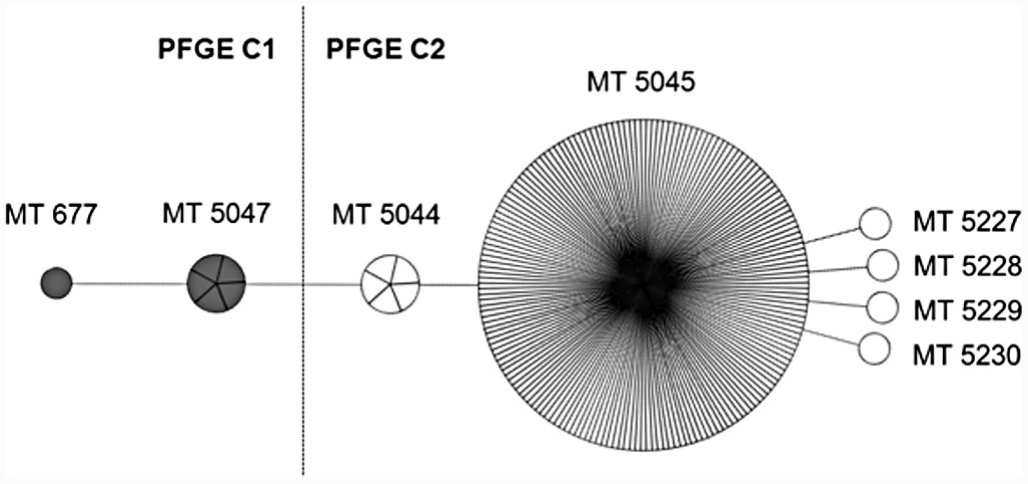
Minimum spanning tree of the 8 different MLVA types found in 194 ST8-MRSA-IV-t304-PVL-negative isolates from 1998–2013. Short lines indicate difference in one VNTR, whereas long lines indicate difference in two VNTRs. The size of the circles corresponds to the number of isolates. MLVA types within PFGE cluster 1 (C1) is marked in grey, whereas MLVA types within PFGE C2 are white.

Distribution of PFGE clusters and MLVA types of the 194 isolates from 1998–2013 are presented in Table 1 and an overview of the MLVA profiles are given in Table 2. Outbreak isolates from three patients were not available for analyses. No MRSA-t304 isolates were detected in 2013. The majority of the isolates (174/194, 90%) were associated with nine outbreaks comprising one hospital and eight nursing homes from 2000–2011, whereas the remaining 20 isolates originated from sporadic cases. PFGE analysis differentiated the isolates into two clusters. MLVA analysis differentiated the isolates into eight MLVA types (Table 1) of which all but MT677 were new types reported here. The MLVA types were specific to only one PFGE cluster each. SID was 0.148 [95% CI 0.079–0.217] for MLVA and 0.060 [95% CI 0.014–0.107] for PFGE. Genetic relationship between MLVA types within the PFGE clusters are presented in Fig 1.

**Table 1:** Distribution of ST8-MRSA-IV-t304-PVL-negative isolates from 1998–2013 according to PFGE and MLVA analyses.

PFGE cluster	MLVA type (MT)	1998	1999	2000	2001–2003	2004	2005	2006	2007	2008	2009	2010	2011	2012	2013*	Total
**C1**	**677**	1														1
	**5047**		1	2								1		1		5
**C2**	**5045**					24	11	46	47	22	7	7	15			179
	**5044**							3				1		1		5
	**5227**							1								1
	**5230**								1							1
	**5228**									1						1
	**5229**											1				1
	**Total**	1	1	2	0	24	11	50	48	23	7	10	15	2	0	194

* Included through April 2013

**Table 2:** Overview of all MLVA types and profiles identified in 194 ST8-MRSA-IV-t304-PVL negative isolates.

	MLVA allele profile							
	V09_01	V61_01	V61_02	V67_01	V21_01	V24_01	V63_01	V81_01
MLVA type (MT)	*sspa*	non-coding	non-coding	non-coding	non-coding	*spa*	non-coding	*coa*
**677**	12	3	3	7	1	10	2	4
**5047**	12	3	3	5	1	10	2	2
**5045**	**12**	**4**	**4**	**5**	**1**	**10**	**2**	**3**
**5044**	12	4	3	5	1	10	2	3
**5227**	12	4	4	5	1	10	2	2
**5230**	12	4	4	5	1	10	1	3
**5228**	12	4	5	5	1	10	2	3
**5229**	12	4	4	3	1	10	2	3

*sspa*: cystein protease; *spa*: Staphylococcal protein A; *coa*: staphylocoagulase; Major clone indicated by profile numbers in bold

PFGE cluster C1 was subdivided into MLVA types MT677 and MT5047 which differed in two VNTRs (Table 2). C1/MT677 was only detected in one sporadic case in 1998 (Table 1). C1/MT5047 caused a small outbreak and was found in a sporadic case in 1999/2000 and reappeared as sporadic cases in 2010 and 2012.

PFGE cluster C2 was subdivided into six new MLVA types. C2/MT5045 consisted of 179/194 (92%) of the isolates and was related to several defined outbreaks in one hospital and seven nursing homes. No new outbreaks were detected after the last appearance of the major outbreak clone C2/MT5045 in 2011. Five closely related variants of MT5045 differing in only one VNTR were identified in 9 patients (Tables 1 and 2, Fig 1). C2/MT5044 was found in 2006 in three patients epidemiologically associated to one of the seven nursing homes with a C2/MT5045 outbreak. This variant also appeared in two sporadic cases in 2010 and 2012. The C2/MT 5227 was also associated with the same nursing home as C2/MT5044. The remaining three MLVA type variants (N = 3 isolates) were identified in three other nursing homes (Table 1).

## Discussion

The MRSA-t304 clone had an endemic-like appearance in Norway, especially in healthcare settings, being reported in 52% of the MRSA isolates affecting nursing homes in Oslo in 2005–2011 [12]. An accurate and discriminatory typing method is important in surveillance and outbreak investigations as outbreaks triggers costly measures and inflict physical and psychological stress and discomfort for the involved individuals.

In this selection of 194 MRSA-t304 isolates spanning over a 15-year time period, MLVA differentiated better between epidemiologically related and unrelated isolates compared to PFGE. Although the SID was higher in MLVA, the difference was not significant and both methods were unable to separate between all epidemiologically unrelated isolates. This may be explained by unknown epidemiological connections between these cases and known outbreaks or that the resolution power of both PFGE and MLVA was too low in this setting due to a highly conserved clone. Although MLVA subdivided the major PFGE cluster C2 into six MLVA types, either methods could differentiate among isolates belonging to eight epidemiologically defined outbreaks in eight different locations appearing from 2004 to 2011. A superior resolution could be obtained using next generation sequencing analyses like whole genome sequencing or single nucleotide polymorphism analysis. However, the lack of easy-to-use pipeline and bioinformatic tools to analyse the large amount of data produced is still a hamper and expensive specialized equipment is needed. Nevertheless, in the future we will attempt to use some of these methods to investigate the level of difference in MRSA-t304 isolates as this would add valuable information in relation to surveillance and outbreak investigations as well as transmission routes and bacterial evolution. Regardless of typing method of choice, it is important that the results are interpreted in the context of all available epidemiological, clinical and demographic data [9].

Interestingly, no new isolates with the dominating outbreak clone C2/MT5045 was identified after 2011 in the region. Although five new MLVA types differing in only one VNTR compared to MT5045 were discovered, only MT5044 was identified in more than one isolate and was the only of these MLVA types that also appeared after 2011. This suggests that either the C2/MT5045 clone is under control, has become extinct or has moved to other geographical regions. Small evolutionary changes within VNTRs are likely to occur within outbreaks of the current size and timespan. The genotyping results indicate that the eight outbreaks may be defined as one large outbreak with branches in eight different locations. This indication cannot be proven epidemiologically as comprehensive data on possible transmission routes like patient movement between locations and healthcare workers with several employments were not recorded. The outbreaks were handled locally by the individual nursing home or hospital and data regarding links between the different geographic locations were not available. In 2006 and 2008 MRSA cohort units were established at two nursing homes in Oslo. This was done to reduce cross-contamination and isolation of patients, and create a good and equitable living environment for persistent carriers of MRSA [13]. This, together with enhanced emphasis on compliance with basic infection control routines and the “search-and-destroy” policy, may likely have led to the apparent reduction or disappearance of the endemic-like C2/MT5045 clone.

A long time span was seen for the C1/MT5047 clone appearing in 1999/2000 and reappearing in 2010 and 2012. This clone was clearly different from the endemic-like clone C2/MT5045 with three VNTRs difference (Table 2) and <80% similarity in PFGE analysis. Appearance of both highly conserved and diverse MRSA-t304 clones emphasize the importance of other genotyping methods in addition to *spa*-typing in surveillance and outbreak investigations especially when dealing with endemic clones and common *spa*-types. This is exemplified by the appearance of another MRSA-t304 clone in the same region but with ST6 appearing in two individuals in 2008–2009 and then seven others in 2011–2012. ST6-MRSA-t304-PVL-negative comprised three MLVA types that clearly differed from the ST8-MRSA-t304-PVL-negative MLVA types and a forth MLVA type was identified in a methicillin sensitive ST6-MSSA-t304 hospital outbreak (PVL not tested, results not shown). ST6 is genetically different from ST8 sharing only two of seven MLST (multilocus sequence typing) alleles. ST6-MRSA-t304 has also been reported in a continuous neonatal ward outbreak in Copenhagen, Denmark from 2011 [7].

Reports on MRSA-t304 are infrequent, but this genotype has been detected in patients on the Caribbean island Martinique, an island with ~500,000 visitors per year mainly from France [4, 14]. International travelling may facilitate spread to and from Europe. It has been reported that returning international travellers with MRSA infections have contracted strains specific to their country of vacation [14–17]. In the present study, the travel status of the infected or colonized individuals was unknown.

This study has limitations. Only regional MRSA cases were included as national data could not be obtained for the complete study period. The movement of patients and staff between hospitals and nursing homes was not monitored and the epidemiological data may be inadequate in some cases and therefore unable to reveal all cross-contamination. However, the isolates were collected from the most populated area in Norway and are believed to be representative for the area of the endemic clone.

Our results support that *spa*-typing and PFGE are not sufficient in surveillance and outbreak investigation of MRSA-t304. We suggest that MLVA can replace PFGE in such settings, but MLVA may not be the optimal analysis method to discriminate between all epidemiologically unrelated isolates. The present study showed that there are clearly different MRSA-t304 MLVA types and confirmed that there was one major outbreak clone from 2004–2011 which now may be under control, has been extinguished or moved to other geographic regions.
